# Sunlight-Induced Coloration of Silk

**DOI:** 10.1186/s11671-016-1506-6

**Published:** 2016-06-14

**Authors:** Ya Yao, Bin Tang, Wu Chen, Lu Sun, Xungai Wang

**Affiliations:** National Engineering Laboratory for Advanced Yarn and Fabric Formation and Clean Production, Wuhan Textile University, Wuhan, 430073 China; Institute for Frontier Materials, Deakin University, Geelong, Victoria 3216 Australia

**Keywords:** Photo-induction, Coloration, Silk fabric, Gold nanoparticles, Light resistance

## Abstract

Silk fabrics were colored by gold nanoparticles (NPs) that were in situ synthesized through the induction of sunlight. Owing to the localized surface plasmon resonance (LSPR) of gold NPs, the treated silk fabrics presented vivid colors. The photo-induced synthesis of gold NPs was also realized on wet silk through adsorbing gold ions out of solution, which provides a water-saving coloration method for textiles. Besides, the patterning of silk was feasible using this simple sunlight-induced coloration approach. The key factors of coloration including gold ion concentration, pH value, and irradiation time were investigated. Moreover, it was demonstrated that either ultraviolet (UV) light or visible light could induce the generation of gold NPs on silk fabrics. The silk fabrics with gold NPs exhibited high light resistance including great UV-blocking property and excellent fastness to sunlight.

**Electronic supplementary material:**

The online version of this article (doi:10.1186/s11671-016-1506-6) contains supplementary material, which is available to authorized users.

## Background

Noble metal nanoparticles (NPs), including gold and silver NPs, have attracted extensive attention due to their localized surface plasmon resonance (LSPR) [1–4]. Conduction electrons around metal NPs locally oscillate at a certain frequency when light interacts with these NPs. The excitation of surface plasmons by light is known as LSPR [5]. The features of LSPR can be controlled by the particle size [5], shape [6], composition [7], external environment [8], and inter-particle spacing [9]. Many strategies have been used to synthesize the noble metal NPs [10]. The particular optical properties from noble metal NPs can produce brilliant and vivid colors, which can be used for textile coloration [11, 12]. In our previous research, various natural fibers including wool, bamboo, ramie, and cotton fabrics were treated with silver and gold NPs to render fibrous materials’ different functions [11–14]. This study focused on treating silk fabrics with noble metal NPs.

Silk fibers have some prominent properties such as good hand feeling, great drapability, and bright luster [15], which distinguish silk from the other fibers. Silk fabrics have been dyed with many traditional dyes, such as acid dyes [16], disperse dyes [17], and reactive dyes [18]. Most studies on dyeing of silk have been focused on washing fastness [19], water consumption [20], and dye sources [21]. However, color fading and high water and energy consumption remain critical issues facing the conventional dyeing methods. We have developed novel coloration methods for silk based on noble metal NPs [22, 23]. Anisotropic silver NPs were prepared and assembled onto silk fibers through electrostatic interaction. In addition, gold NPs were in situ synthesized on silk by heating, realizing the coloration of silk fabrics. Different from the previous works, the present coloration strategy used sunlight as a green energy resource and a water-saving concept to achieve vivid colors on silk fabrics.

Herein, in situ synthesis of gold NPs on the silk fabrics was achieved through a sunlight-induced process. The as-prepared gold NPs endow fabrics with bright colors and excellent photo-stability due to the LSPR properties, as well as strong ultraviolet (UV) resistance. The key factors including gold ion concentration, pH value, irradiation time, and light source were investigated. The morphology and structure of the treated silk fibers were also observed. The properties of the treated silk fabrics including light fastness, washing fastness, and rubbing fastness were examined. Moreover, water-saving coloration method based on sunlight induction was exploited.

## Methods

### Materials

Tetrachloroauric(III) acid (HAuCl_4_·3H_2_O, >99 %) was purchased from Sigma-Aldrich. All chemicals were analytic grade reagents and used without further purification. Plain weave silk fabrics, with a weight of 67 g/m^2^ and a density of 59 threads/cm in the weft direction and 107 threads/cm in the warp direction, were purchased from a local retailer. They were used without pretreatment.

### Instruments

Ultraviolet-visible (UV-vis) reflectance absorption spectra were obtained from a Varian Cary 5000UV-VIS-NIR spectrophotometer with a diffuse reflectance accessory (DRA-2500). The morphologies of fibers were observed on a Supra 55 VP field emission scanning electron microscope (SEM). A Datacolor Spectraflash SF600 Plus-CT spectrophotometer was used for the measurement of color strength (K/S). A YG902 Fangyuan UV measurement system was used for testing the UV-blocking properties. The light irradiation test was conducted with a Suntest instrument (SUNTEST XLS+ from Atlas Material Testing Technology LLC). The temperature of the coloration process was monitored by an instrument equipped with needle-type temperature probes (from ICT SFM, a sap flow meter produced by ICT international Pty. Ltd.). X-ray diffraction (XRD) data were collected from fabrics on a Panalytical X’Pert Powder instrument using a CuKa radiation source (*λ* = 1.54181 Å). Rubbing color fastness was tested by a Gellowen G238BB electronic crockmeter instrument.

### In Situ Synthesis of Gold NPs on Silk Fabrics

Silk fabrics were washed with deionized water and immersed in HAuCl_4_ aqueous solution with different concentrations (0.2, 0.3, 0.4, 0.5, 0.6, and 0.7 mM). The mass ratio of HAuCl_4_ solution to silk fabric was 100. The solutions containing silk fabrics were shaken for 15 min at room temperature before they were placed under the Suntest instrument for irradiation. After irradiated for 2 h, the silk fabrics were taken out from reaction solution and rinsed with running deionized water. Subsequently, the treated fabrics were dried at room temperature. The treated silk fabrics corresponding to 0.2, 0.3, 0.4, 0.5, 0.6, and 0.7 mM of HAuCl_4_ were denoted as GS-02, GS-03, GS-04, GS-05, GS-06, and GS-07. Unless otherwise stated, the irradiation time and the light power for different samples were 2 h and 250 W, respectively.

### XRD Measurement

Silk fabrics were stuck to glass slides for XRD characterization. XRD analysis of silk fabrics was performed on the Panalytical X’Pert Powder instrument at a voltage of 45 kV and a current of 20 mA. XRD data were collected with 2*θ* range of 10°–80° and a scanning rate of 2°/min at a step of 0.01°.

### Color Fastness Test to Sunlight

The gold NP-treated silk fabrics (8 × 4 cm) corresponding to different concentrations of HAuCl_4_ were stapled with cardboard, and a part (4 × 4 cm) of the sample was covered with cardboard and aluminum foil. After that, the samples were exposed under simulated sunlight in the Suntest instrument for 65, 130, and 195 h at 600 W. The color differences were measured using the Datacolor Spectraflash SF600 Plus-CT spectrophotometer.

### Color Fastness Test to Washing

The Australian Standard AS 2001.4.15—2006 was followed in tests of the washing fastness of the silk fabrics treated with gold NPs. The washing process lasted for 45 min at 50 °C with the ECE reference detergent in a dyeing machine (Ahiba IR Pro Dyeing Machine). The silk fabrics after washing were dried for 15 min at 50 °C in an oven. The color difference (Δ*E*) of silk fabrics was measured by a spectrophotometer before and after washing. Gray scale was assessed using Δ*E* according to the Australian Standard AS 2001.4.A05—2004. A small Δ*E* value means small change in color.

### Color Fastness to Rubbing

The dry and wet rubbing color fastnesses of treated silk fabrics were evaluated according to the Australian Standard AS 2001.4.3—1995. The fabrics colored with gold NPs were rubbed using an undyed cotton cloth. The staining of the cotton cloths were assessed using the standard gray scale for staining. Both dry and wet rubbing fastness tests were performed.

## Results and Discussion

### Coloration of Silk Fabrics via In Situ Synthesis of Gold NPs

**Fig. 1:**
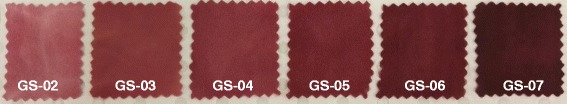
Optical images of the silk fabrics treated with different concentrations of HAuCl_4_ solutions

**Fig. 2:**
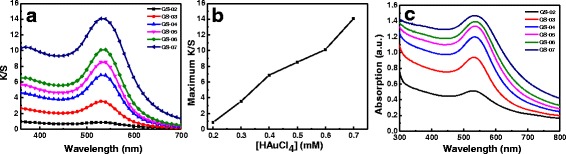
**a** K/S curves of the silk fabrics treated in solutions with different concentrations of HAuCl_4_ after irradiation. **b** Plot of peak K/S values in **a** as a function of HAuCl_4_ concentration. **c** UV-vis absorption spectra of the treated silk fabrics corresponding to **a**

Figure 1 shows the silk fabrics treated in the presence of HAuCl_4_ solution under simulated sunlight. As can be seen, the silk fabrics changed to red or brownish red from white. The color differences of silk fabrics reveal that gold NPs were synthesized in situ on silk fabrics by irradiation of simulated sunlight. The color of fabric was red when the concentration of HAuCl_4_ was 0.2 mM and became darker as the concentration of gold ions increased. The K/S curves of the silk fabrics were measured to investigate the influences of gold ion concentration on colors of the treated silk fabrics (Fig. 2a). The peak K/S values all appeared at the wavelength around 530 nm. The peak K/S values increased with an increase in the concentration of HAuCl_4_. The plot of peak K/S values as a function of HAuCl_4_ concentration is shown in Fig. 2b. The peak K/S value increased to 14.08 from 0.90 when the concentration of HAuCl_4_ solution increased to 0.7 from 0.2 mM. These results indicate that the color of silk fabrics treated with gold NPs can be tuned by controlling the concentration of HAuCl_4_. It was mentioned that the color of the silk fabrics treated with gold NPs was related to the LSPR properties of gold NPs [22, 23]. To gain insights into the detailed optical features of silk fabrics with gold NPs, the UV-vis reflectance absorption spectra of the treated silk fabrics were recorded (Fig. 2c). The fabrics treated with 0.2 mM of HAuCl_4_ (GS-02) displayed an absorption band at 530 nm, assigned to the LSPR band of gold NPs [22], which suggests that the gold NPs were in situ synthesized on silk fabrics by the induced effect of sunlight. The UV-vis absorption band of the treated fabrics red-shifted to 540 nm as the concentration of HAuCl_4_ increased to 0.7 from 0.2 mM. The different absorption spectra of the treated fabrics may be due to the differences in morphologies of gold NPs in situ synthesized on silk fibers.

To check the effect of natural sunlight on the synthesis of gold NPs, the irradiation of samples was implemented outdoor under natural sunlight. Similar to the case of simulated sunlight, the photo-induction of natural sunlight produced bright colors including red and brownish red on silk fabrics (Additional file 1: Figure S1), which reveals that the photo-induced coloration of silk with gold NPs can be realized using natural sunlight.

### Influences of Irradiation Time and pH Value

**Fig. 3:**
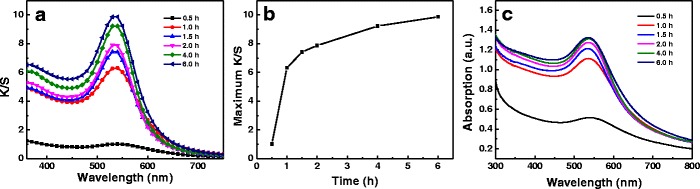
**a** K/S curves of the silk fabrics with gold NPs corresponding to 0.5 mM of HAuCl_4_ at different irradiated time. **b** Plot of peak K/S value as a function of irradiation time. **c** UV-vis reflectance absorption spectra of the treated silk fabrics corresponding to **a**

To observe the effect of irradiation time on the coloration of silk, the K/S curves of the treated silk fabrics were obtained at certain interval during simulated sunlight induction process (Fig. 3a). The wavelength at maximum K/S value did not vary visibly as the irradiation time prolonged. The peak K/S value of treated silk fabrics increased as the irradiation time increased (Fig. 3a). The plot of maximum K/S values versus irradiation time is depicted in Fig. 3b. The K/S values of the treated fabrics increased dramatically when the irradiation time was increased to 1.0 from 0.5 h, suggesting the in situ synthesis rate of gold NPs on silk was high at the beginning of light induction. It may be inferred that the initial period (≤1 h) of light irradiation is a fast growth stage of gold NPs. The K/S values of the treated fabrics increased very slowly after 2 h irradiation by simulated sunlight (Fig. 3b), implying that the synthesis of gold NPs nearly completed. In addition, UV-vis reflectance absorption spectra of the treated silk fabrics showed the same trend as the K/S curves as irradiation time varied (Fig. 3c). The LSPR band assigned to gold NPs was found when reaction system was irradiated for 0.5 h, which demonstrated that gold NPs were produced in a short time of irradiation.

**Fig. 4:**
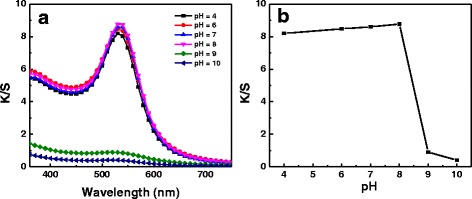
**a** K/S curves of the silk fabrics with gold NPs corresponding to 0.5 mM of HAuCl_4_ at different pH values. **b** Plot of peak K/S value as a function of pH value

In the present study, the influence of pH value on the coloration of silk was investigated. The pH values of HAuCl_4_ solution at different concentrations were tested to be 5.0~6.5 [24]. Figure 4a displays the K/S curves of silk fabrics treated at different pH values. Bright colors could be obtained on the silk fabrics when pH values of the reaction system were in the range of 4~8. Nevertheless, no obvious colors were seen on the silk fabrics as the pH value increased to 9. The peak K/S of the treated fabrics with different pH values was plotted as a function of pH value (Fig. 4b). The peak K/S value changed slightly even though the pH value was changed to 8 from 4. The results revealed that acidic condition favored the in situ synthesis of gold NPs on silk fabrics.

### Characterization of Silk Fabrics Treated with Gold NPs

**Fig. 5:**
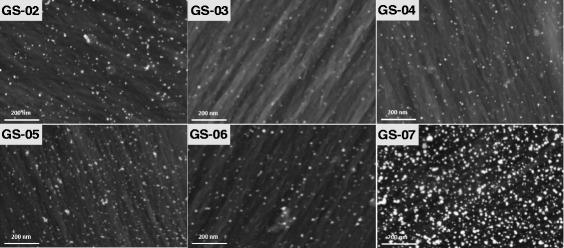
SEM images of the silk fabrics treated with gold nanoparticles

The LSPR properties of noble metal NPs are sensitive to their shape [25] and size [6]. Figure 5 shows the SEM images of the treated silk fabrics. The NPs can be seen clearly in the SEM images, revealing that the gold NPs were obtained on the fiber surface. Most of the gold NPs were spherical and dispersed homogeneously on the surface of silk fibers, although a few aggregates of NPs were also present. The sizes of the gold NPs were measured to be 16.9 ± 1.2, 24.1 ± 1.7, 23.0 ± 2.1, 20.6 ± 1.2, 19.9 ± 1.3, and 28.4 ± 1.6 nm corresponding to GS-02~GS-07, respectively. The size of the NPs for GS-07 was the largest, and the anisotropic morphologies appeared in addition to spherical shape, which resulted in the differences in absorption spectrum of the treated fabric (GS-07) (Fig. 2c). The silk fabrics treated with gold NPs displayed bright colors because of the LSPR property of NPs.

**Fig. 6:**
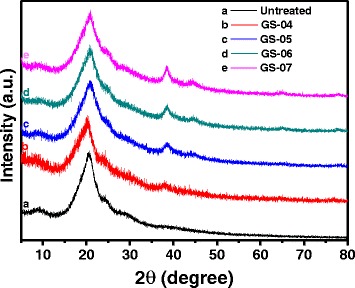
XRD spectra of the untreated and treated silk fabrics

XRD measurements were performed to investigate the crystalline structure of the silk fabrics after treatment. The XRD peaks at 8.75°, 18.54°, 20.49°, and 24.41° were seen in the pattern of untreated silk fabrics (curve a in Fig. 6), which are assigned to crystallinity of silk structure [26]. A new peak at 38.46° appeared in the XRD pattern of the silk fabric that was treated in 0.4 mM of HAuCl_4_ solution (GS-04). This peak around 38.46° increased in intensity, and another peak around 44.25° arose as the initial HAuCl_4_ concentration increased to 0.6 mM (GS-06, curve d in Fig. 6). The XRD peaks at 38.46° and 44.25° are attributed to the crystallinity of gold NPs [27]. The intensity of the two peaks assigned to gold increased with the concentration of HAuCl_4_, which may be due to an increased amount of gold nanocrystals on silk fabrics. However, comparing the XRD patterns of silk fabrics before and after treatment, no visible changes in XRD peaks of silk were found, implying that the light irradiation during synthesis of gold NPs did not change the crystal structure of silk fibers.

### Properties of Gold NP-Colored Silk Fabrics

**Fig. 7:**
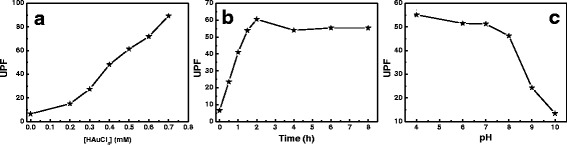
**a** UPF values of silk fabrics treated with gold NPs as a function concentration of gold ions. **b** UPF values of silk fabrics treated with 0.5 mM HAuCl_4_ at different irradiation time. **c** Plot of UPF values of silk fabrics treated with 0.5 mM HAuCl_4_ as a function of pH value

In this study, the UV-blocking properties of the treated silk fabrics were tested. The average transmittance values of the treated silk fabrics decreased when the concentration of HAuCl_4_ increased, which indicates that the gold NPs advanced the UV-blocking properties apparently. UV protection factor (UPF) is an important factor for evaluating the ability of fabrics to resist UV light from passing through fabrics and touching the skin [28, 29]. The UPF value of the untreated silk fabrics was 6.46 (Fig. 7a). It is seen that the UPF values of treated silk fabrics increased significantly with an increase in HAuCl_4_ concentration (Fig. 7a). The UPF of the treated fabrics increased to 89.3 corresponding to 0.7 mM of HAuCl_4_ (Fig. 7a). The UV-blocking ability of silk fabrics increased with gold content in silk. Additionally, the influences of irradiation time and pH value on the UV protection property of silk fabrics were examined. The UPF value of silk fabric increased with irradiation time at the beginning of sunlight-induced process and changed slightly after irradiated for 2 h (Fig. 7b), indicating that the reasonable irradiation time is 2 h for obtaining the optimal UV-blocking property of silk fabrics. The UPF value of the treated silk fabrics decreased dramatically as the pH value was more than 8 (Fig. 7c), which indicates that acid treatment condition is beneficial for UV protection of silk fabrics with gold NPs. These results manifest that the in situ synthesized gold NPs impart great UV protection feature to silk fabrics, enhancing the function of silk fabrics.

**Fig. 8:**
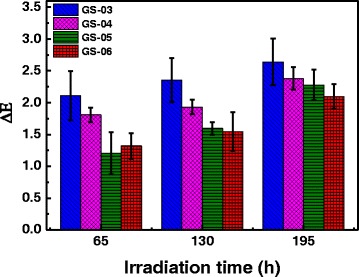
Color differences (∆*E*) of silk fabrics treated with different concentrations of HAuCl_4_ solutions after exposure to simulated sunlight with the power of 600 W for different periods

Many existing dyes fade severely upon exposure to sunlight for a prolonged period [30]. Thus, avoiding photo-fading of colored textiles is a critical challenge in industry and has been extensively investigated for nearly two centuries. Color fading is attributed mainly to the photo-degradation of the chemical structures of conventional dyes [31–33]. The structure of the textiles will be damaged during the irradiation of sunlight, and it would lose the protection of the skin from the long period of exposure to the sunlight. The NPs on the treated silk fabrics were expected to be stable even after exposure to the sunlight for a long time. The treated silk fabrics were irradiated with the simulated sunlight (600 W) for different periods (65, 130, and 195 h), and color differences were measured after exposure to simulated sunlight (Fig. 8). The average color difference (∆*E*) of GS-03 was 2.1 after exposure to the sunlight for 65 h, while the color differences of GS-04, GS-05, and GS-06 were less than 2 after 65 h of exposure. It is suggested that the higher gold content provides stronger light resistance ability to fabrics. The color differences of treated silk fabrics increased slightly with exposure time. The ∆*E* value of GS-03 was lower than 2.7 even if the silk fabric was exposed to the simulated sunlight for 195 h. The results demonstrate that the gold NP-treated silk fabrics have excellent color fastness to sunlight. The gold NPs on silk fabrics were in situ synthesized by photo-induction of sunlight, and their morphologies would not vary under prolonged sunlight irradiation. The stable morphologies of gold NPs ensure the stability of colors from LSPR optical features of NPs. Thus, it can be deduced that the photo-induced synthesis of gold NPs based on sunlight is favorable of producing the dyed textiles with high color fastness to light. Importantly, the existence of gold NPs protects silk fabrics from damage or degradation from sunlight irradiation. An untreated (pristine) silk fabric changed color noticeably when it was irradiated for 195 h under simulated sunlight (600 W) (Additional file 1: Figure S2). The color difference of silk fabrics before and after exposure was 11.8, much higher than those of the gold NP-treated fabrics, which indicates that gold NPs play a significant role in protection of silk fabric from damage by sunlight.

**Fig. 9:**
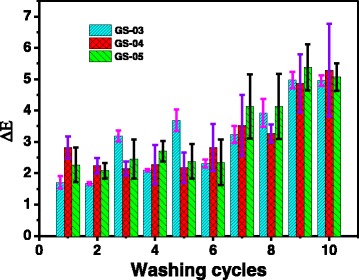
Color differences (∆*E*) of the treated silk fabrics after different washing cycles

The color fastness to washing is also very important to the utilization of the dyed textile products [34]. We evaluated the washing color fastness of the silk fabrics treated with gold NP. The treated silk fabrics were washed for ten times at 50 °C for 45 min each time with the ECE reference detergent. The average color differences of GS-03, GS-04, and GS-05 after first washing cycle were 1.7, 2.8, and 2.3, respectively (Fig. 9). The gray ratings of color fastness according to color differences were evaluated to be 4, 3.5, and 3.5 for GS-03, GS-04, and GS-05, respectively. Although increasing washing cycles increased the color differences of the treated silk fabrics, the ∆*E* values tended to stabilize around 5 after being washed for nine times (Fig. 9).

Fabrics are often rubbed against other surfaces, and color change during the abrasion process should be minimized. In the present study, the color fastness to rubbing of the samples GS-04 and GS-06 was tested. The dry rubbing color fastness of the gold NP-treated silk fabrics was rated as 5 for both GS-04 and GS-06. And the rating of the wet color fastness of the fabrics with gold NPs was assessed to be 5. The staining of the cotton cloths was assessed to be 4–5 for both GS-04 and GS-06 under wet and dry conditions. The results demonstrate that the silk fabrics colored with gold NPs exhibited good color fastness to rubbing (dry or wet).

### Investigation of Light Source and Water Consumption

It has been reported that the gold NPs could be in situ synthesized on silk fabrics by heating [22]. Is it possible for the heat effect from sunlight to result in the synthesis of gold NPs during the photo-induced process? To clarify this point, the time-resolved temperature of reaction solution during the simulated sunlight-induced process was recorded with a thermal sensor. The curve of temperature as a function of irradiation time was plotted in Additional file 1: Figure S3. The temperature of reaction solution increased to ~31.5 from 23.3 °C (room temperature) within 90 min and kept almost unchanged during the irradiation process. In order to further inspect the effect of heat from sunlight, the solution containing silk fabric (0.2 g) and HAuCl_4_ solution (0.5 mM, 40 mL) was heated for 2 h at 40 °C (higher than 31.5). The color of silk fabric after heating did not change observably. Besides, the flask containing silk fabrics and HAuCl_4_ solution was wrapped with an aluminum foil to shade the light and subsequently placed in the Suntest instrument. No visible color differences of the silk fabric were found even though the flask was irradiated for 5 h under simulated sunlight at 250 W. These results verify that the heat from sunlight induction cannot have gold NPs in situ synthesized on silk fabrics.

The effect of light source on the synthesis of gold NPs on silk was also investigated. An UV lamp was employed to irradiate reaction system to inspect the role of UV light on the in situ synthesis of gold NPs. The emission spectrum of the UV lamp is shown in Additional file 1: Figure S4, with two main emission bands in the range of 340–450 nm. Additional file 1: Figure S5 displays optical images of silk fabrics after being irradiated under UV lamp for 2 h in the presence of 0.4 and 0.5 mM HAuCl_4_. The colors of fabrics turned out to be red after UV light irradiation, which suggests that in situ synthesis of gold NPs can be realized by UV light. Furthermore, different optical filters were used to cover the reaction solution during irradiation process to find out the influences from the different parts of sunlight. The transmission spectra of the optical filters are shown in Additional file 1: Figure S6. Red silk fabric was obtained when it was irradiated by simulated sunlight, covered with the optical filter (Additional file 1: Figure S6a) that blocks the light with wavelength less than 385 nm. The cutoff of most UV light from simulated sunlight did not prevent the in situ synthesis of gold NPs on silk fabrics. The filter in Additional file 1: Figure S6b only allows the passage of light in the region of 550–630 nm. The color of silk fabric irradiated with the filter in Additional file 1: Figure S6b was light red, implying that the visible light at long wavelength with low energy can still induce the synthesis of gold NPs on silk, although the amount of synthesized gold NPs was low. It is inferred that not only UV light but also visible light could induce the synthesis of gold NPs on silk.

**Fig. 10:**
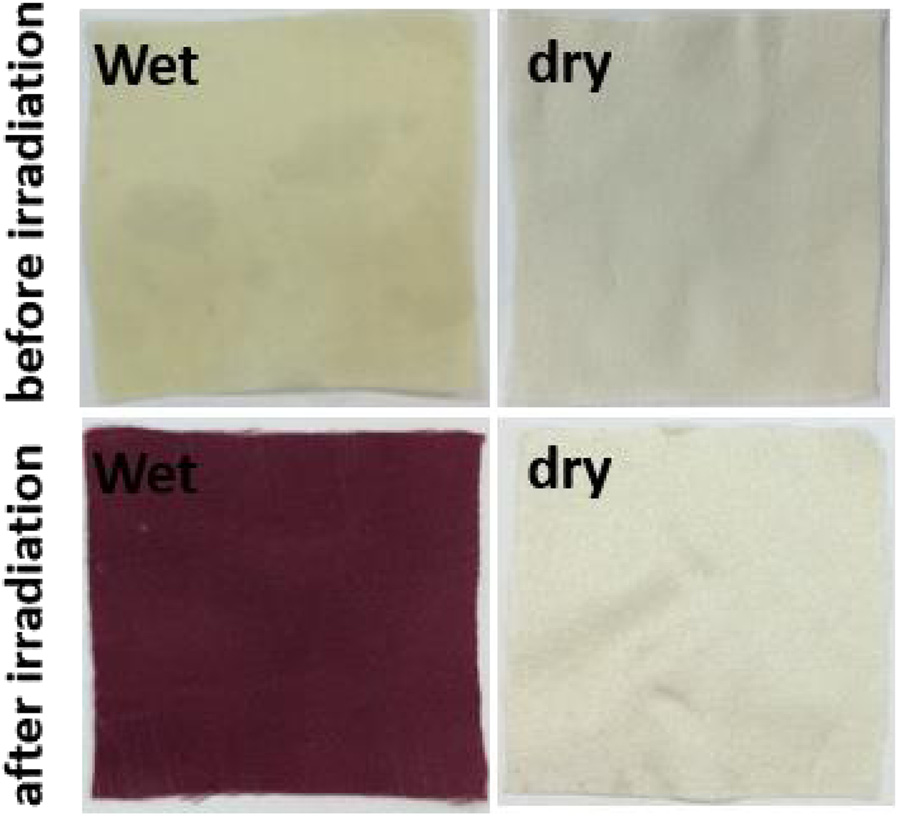
Wet and dry silk fabrics adsorbing gold ions taken out from solution before and after irradiation of simulated sunlight

Water consumption and water pollution are critical issues for the textile dyeing industry [35, 36]. In the present study, we took the water consumption into consideration and tried to realize water-saving coloration of silk based on gold NPs. The silk fabrics were immersed in the HAuCl_4_ solutions and shaken for 30 min in the room temperature. And then, the fabrics adsorbing gold ions were taken out from the solution and irradiated by simulated sunlight. As can be seen in Fig. 10, the wet silk fabrics became red after 2 h of irradiation, implying that gold NPs were produced even when the silk fabrics were not in solution, whereas the silk fabric adsorbing gold ions after drying did not change notably in color under light irradiation (Fig. 10). The results indicate that a humid environment is essential for the in situ synthesis of gold NPs on silk fabrics through photo-induction. It is worth noting that silk fabrics out of aqueous solution could be colored by gold NPs as long as the fabrics were kept wet. Moreover, the water left in solution could be reused for the next coloration cycle. Thus, the water consumption for the present coloration method is very low, which facilitates the development of environment-friendly dyeing technology of textiles.

### Patterning Application of Silk Fabrics Treated with In Situ Synthesized Gold NPs

**Fig. 11:**
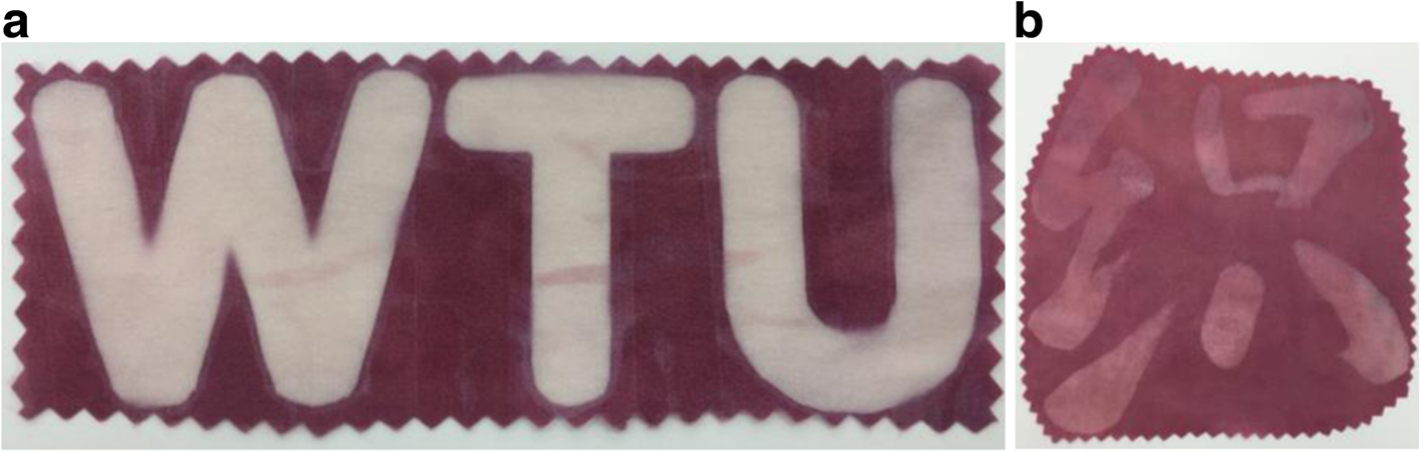
Photographs of the gold NP-colored silk fabrics with different patterns: **a** a word “WTU” and **b** a Chinese character meaning “weave”. The concentrations of HAuCl_4_ corresponding to **a** and **b** were 0.5 and 0.3 mM, respectively

The realization of coloration of wet silk fabrics by sunlight induction inspired us to develop patterning of silk based on light irradiation. A transparent plastic sample bag containing wet silk with gold ions was covered by a mask with a pattern (WTU). A red silk fabric with “WTU” pattern was obtained after light irradiation (Fig. 11a). The parts of silk without irradiation did not produce gold NPs though gold ions were adsorbed there, resulting in the transfer of pattern from mask to fabric. In addition, the gold NP coloration method based on sunlight induction provides a more convenient route to make patterns on silk, without preparing masks. A Chinese character was written on a sample bag containing wet silk using a Chinese brush. The black ink blocked the transmission of light as a mask and the pattern of the Chinese character displayed on silk fabrics after 2 h of irradiation. Subsequently, the wet silk with “white” pattern was transferred into new sample bag and irradiated again by simulated sunlight for 1 h. The colored pattern was achieved on silk fabrics with a light-blocking pattern by two steps of irradiation (Fig. 11b). Owing to convenience and effectiveness of photo-induction method, the present coloration technology has potential for pattern and fashion design-related applications.

## Conclusions

The in situ synthesis of gold nanoparticles (NPs) on silk fabrics was realized by the induction of sunlight. The as-synthesized gold NPs endowed silk fabrics with bright colors due to localized surface plasmon resonance (LSPR) of metal NPs. The K/S of the treated silk fabrics could be tuned by controlling the concentration of gold ions in reaction system. Acid condition favored the sunlight-induced synthesis of gold NPs. The colored silk fabrics exhibited great color fastness to sunlight. Significantly, the treatment with gold NPs rendered excellent UV protection ability to silk fabrics. The photo-induced coloration based on gold NPs was also feasible under a humid condition without large water consumption, which not only provides a novel water-saving dyeing technology but also offers an effective route to achieve pattern dyeing of fabrics. Combination of sunlight-induced coloration method and surface modification of fibers will facilitate the development of multi-functional textiles.

## Additional file

Additional file 1: The file contains supplementary Figures S1–S6. (DOCX 1.63 MB)
