# Supplementary material for: Sunlight-Induced Coloration of Silk
Source: Nanoscale Res Lett. 2016 Jun 14;11:293. doi: 10.1186/s11671-016-1506-6 (PMC4905859; doi:10.1186/s11671-016-1506-6)
Supplement: Additional file 1: — The file contains supplementary Figures S1–S6. (DOCX 1.63 MB) [file 11671_2016_1506_MOESM1_ESM.docx]

Additional file 1

**Sunlight-Induced Coloration of Silk**

Ya Yao^1,2^, Bin Tang^1,2*^, Wu Chen^1^, Lu Sun^1,2^, Xungai Wang^1,2 *^

^1^National Engineering Laboratory for Advanced Yarn and Fabric Formation and Clean Production, Wuhan Textile University, Wuhan 430073, China

^2^Institute for Frontier Materials, Deakin University, Geelong, Victoria 3216, Australia

^*^ Correspondence: bin.tang@deakin.edu.au; xungai.wang@deakin.edu.au

**Figure S1.** Optical images of the silk fabrics after being treated under natural sunlight with different concentrations of HAuCl_4_: **a** 0.3 mM, **b** 0.4 mM, **c** 0. 5 mM, **d** 0.6 mM, **e** 0.7 mM. The irradiation site was located at 38°11´48´´ south longitude and 144°17´44´´ east longitude.

**Figure S2.** Photograph of pristine silk fabric **a** before and **b** after being irradiated for 195 h by simulated sunlight at 600 W.

**Figure S3.** Evolution of the temperature of the reaction system corresponding to 0.5 mM of HAuCl_4_ with irradiation time under simulated sunlight at 250 W.

**Figure S4.** Emission spectrum of UV lamp used in the present study.

**Figure S5.** Photographs of silk fabrics with **a** 0.4 and **b** 0.5 mM of HAuCl_4_ after being irradiated for 2 h under UV light.

**Figure S6.** Transmission spectra of optical filters used to cover the samples under simulated sunlight. Insets: silk fabrics corresponding to different filters after being irradiated for 2 h by simulated sunlight at 250 W.
